# Clinical and Electrophysiological Characterization of Diabetic Neuropathy in a Sub‐Saharan African Cohort

**DOI:** 10.1111/jns.70021

**Published:** 2025-05-09

**Authors:** Samuel Eric Chokote, Gaelle Lemdjo, Juan Francisco Idiaquez Rios, Aurelien Tejiozem Anakeu, Leonard Ngarka, Leonard N. Nfor, Michel K. Mengnjo, Wepnyu Y. Njamnshi, Herman Nestor Tsague Kengni, Ruth Joelle Ngongang, Gilles Simeni, Lylian Piameu, Alain Balla Nkonda, Faustin Yepnjio, Godwin Y. Tatah, Umapathi N. Thirugnanam, Alfred Kongnyu Njamnshi

**Affiliations:** ^1^ Neurology Department Jamot Hospital Yaoundé Cameroon; ^2^ Brain Research Africa Initiative Yaoundé Cameroon; ^3^ Brain Research Africa Initiative Geneva Switzerland; ^4^ Endocrinology Department Jordan Hospital Yaoundé Yaoundé Cameroon; ^5^ Division of Neurology Toronto Ellen and Martin Prosserman Centre for Neuromuscular Diseases Toronto Canada; ^6^ Department of Medicine University Health Network, Toronto General Hospital Toronto Canada; ^7^ Company Criystallise Limited London UK; ^8^ Neurology Department Yaoundé Central Hospital Yaoundé Cameroon; ^9^ Neuroscience Lab, Faculty of Medicine and Biomedical Sciences The University of Yaoundé I Yaoundé Cameroon; ^10^ Global Brain Health Institute, Neuroscience Center, University of California San Fransisco California USA; ^11^ Faculty of Medicine and Pharmaceutical Sciences The University of Douala Douala Cameroon; ^12^ Laboratory Department Jordan Medical Services Yaoundé Cameroon; ^13^ Neurology Department Saint Nazaire Hospital Canter Saint Nazaire France; ^14^ National Neuroscience Institute Singapore Singapore; ^15^ Singapore National Eye Centre Singapore Singapore

**Keywords:** diabetic neuropathy, distal symmetric polyneuropathy, small fibre neuropathy, treatment‐induced neuropathy of diabetes

## Abstract

**Background:**

Diabetic neuropathy (DN) is the most frequent complication of diabetes mellitus, contributing to increased morbidity and mortality. Previous clinical studies on DN in sub‐Saharan Africa (sSA) have used purely clinical approaches, potentially underestimating the true magnitude of this disease. This study was designed to determine the prevalence of definite diabetic neuropathy and describe the different subtypes using objective small and large fiber function measures.

**Methods:**

This was a hospital‐based cross‐sectional study that included diabetes and prediabetes patients, followed up at Jordan Medical Services, Yaoundé, Cameroon, between March 2022 and February 2023. The “Toronto Clinical Neuropathy Score” and “Douleur Neuropathique en 4” questionnaires were used for clinical evaluation. Autonomic symptoms were equally recorded. Nerve conduction studies and Sudoscan were used for electrophysiological assessments of large and small fibre functions.

**Results:**

Eighty‐four participants were included; 91.7% had type 2 DM, 2.4% had type 1 DM, and 6% had glucose intolerance. DN was found in 73/84 (86.9%). Diabetic sensorimotor polyneuropathy (DSP) was the most frequent subtype (63.8%), followed by diabetic autonomic neuropathy (40.5%), mononeuropathy (36.9%), asymmetric axonal sensory neuropathy (4.8%) and treatment‐induced neuropathy of diabetes (TIND) in 1.2% of patients. The prevalence of large and small fibre neuropathies was 38.1% and 25.0%, respectively.

**Conclusion:**

The prevalence of DN and specifically DSP in our study was higher than previously described in African literature. We identified subtypes never before reported in sSA, mainly small fibre neuropathy and TIND. This may have management and policy implications.

**Abbreviations**
BMI: Body Mass Index
CAN: Cardiac Autonomic Neuropathy
CMAP: Compound Muscle Action Potential
CV: Conduction Velocity
DM: Diabetes Mellitus
DN: Diabetic Neuropathy
DN4: Douleur Neuropathique en 4 points
DSP: Diabetic Sensorimotor Polyneuropathy
EDX: Electrodiagnostic studies
ESC: Electrochemical Skin Conductances
IENFD: Intra Epidermal Nerve Fibre Density
LFN: Large fibre Neuropathy
SFN: Small fibre Neuropathy
SNAP: Sensory Nerve Action Potential
sSA: sub‐Saharan Africa
TCNS: Toronto Clinical Neuropathy Score
TIND: Treatment Induced Neuropathy of Diabetes
VPT: Vibration Perception Threshold

## 1 Introduction

Diabetes Mellitus (DM) is a significant global health challenge, regarded as a modern pandemic. Africa faces a particularly high burden, with 59.7% of DM cases undiagnosed and 73.1% of deaths occurring in individuals under 60 years old [1]. Diabetic neuropathy (DN) is the most common and earliest chronic complication of DM, with a prevalence in Africa reaching 46%, surpassing the global rate of 33% [2, 3, 4]. Over the past two decades, sub‐Saharan Africa (sSA) has experienced a significant rise in non‐communicable diseases (NCDs), such as diabetes, primarily due to lifestyle changes and environmental factors. Accurate epidemiological data are essential to develop effective prevention and control strategies for these conditions.

DN is a heterogeneous disease, with distal symmetric sensorimotor polyneuropathy (DSP) as the predominant subtype. Classification of DN requires evaluation across multiple dimensions, including clinical symptoms, electrodiagnostic studies (EDX), quantitative sensory testing, and autonomic function testing [5]. The Toronto Consensus outlines minimum criteria for diagnosing DSP, recommending EDX to improve diagnostic precision and detect subclinical DSP [6, 7].

African studies have relied mainly on clinical assessments alone, leading to potential overestimation and variable accuracy of DN diagnoses. A systematic review in 2020 found that all 23 studies included used clinical‐only diagnostic methods, with subsequent studies in Tanzania, Uganda, and Ethiopia following a similar approach [4, 8, 9, 10]. While one study in Ethiopia used nerve conduction, it lacked detailed reporting on DSP parameters and results [11].

The full spectrum of DN and the prevalence of DSP in Africa still need to be explored, impacting the understanding of its prognosis across subtypes. Evidence suggests that small fibre neuropathy (SFN), often appearing early, may be reversible, and ethnic variability in small fibre function has been observed [12, 13].

The limited availability of neurophysiological testing in sSA, as evidenced by the scarce accessibility of nerve conduction studies and the paucity of intraepidermal nerve fibre density (IENFD) data [14], underscores the need to explore validated point‐of‐care diagnostic alternatives for small fiber neuropathy (SFN) in this region.

This pilot study aims to provide a clearer assessment of DN prevalence and subtype distribution in an African cohort, using objective small and large fibre measures, aligning with the World Federation of Neurology's mission to advance clinical research and practice standards across the continent [15]. Recent evidence suggests that factors beyond hyperglycemia severity and duration underlie the pathophysiology of DN. Hence, we included patients with IGT to record a more comprehensive picture of the spectrum of DN.

## 2 Participants and Methods

### 2.1 Study Site, Design, and Participants

This was a hospital‐based observational, cross‐sectional study at the Endocrinology and Neurology departments of Jordan Medical Services in Yaoundé, Cameroon. Participants were recruited consecutively between March 2022 and February 2023 and were included if they met the American Diabetes Association 2021 criteria for diabetes or prediabetes [16], either already followed up or recently diagnosed. All subjects presenting with an acute diabetic complication (hypoglycemic, hyperosmolar or ketoacidosis coma), in the acute phase of a cerebrovascular or cardiovascular event (stroke, myocardial infarction) or who could not undergo a complete neurophysiological testing (limb amputation, extensive wounds) were excluded from the study.

### 2.2 Procedure

Participants were mostly encountered at the endocrinology and diabetology unit of Jordan Medical Services. Only those who gave consent were included. The first clinical evaluation was carried out by the endocrinologist (GL). This included a collection of sociodemographic data, history of diabetes age and circumstances of diagnosis; type; acute and chronic complications; comorbidities, mainly hypertension, dyslipidemia, smoking, alcoholism, and physical inactivity. Symptoms suggestive of DN were recorded, including pain, paresthesias, numbness, and muscle weakness with their topographic distribution, duration, and mode of onset. Symptoms of dysautonomia were equally searched for (gastroparesis, postprandial nausea or vomiting, chronic constipation or diarrhea, dysuria, urgency or urinary incontinence, chronic urine retention, erectile dysfunction, anhydrosis or hyperhidrosis and orthostatic dizziness). During this first visit, physical examination focused on hemodynamic and anthropometric parameters (supine and orthostatic blood pressure, weight, height, body mass index, waist circumference), palpation of pulses at the four limbs, evaluation of both legs in search of diabetic foot ulcers, and the monofilament test using the 10 g Semmes‐Weinstein monofilament.

The neurological evaluation, conducted by a neurologist (SEC) blinded to prior results, assessed global and segmental muscle strength using the Medical Research Council (MRC) scale, myotatic reflexes, muscle amyotrophy, small fibre function (pinprick sensation and temperature perception), and large fibre sensation (proprioception and vibration perception threshold). Cranial nerve palsies were documented, and clinical data were used to complete the Toronto Clinical Neuropathy Score (TCNS) and “Douleur Neuropathique en 4 points” (DN4) questionnaire. Vibration perception threshold (VPT), Electrodiagnostic studies (EDX) and Electrochemical Skin Conductance (ESC) were performed.

As part of routine follow‐up, blood and urine testing were performed in all patients to assess glycated hemoglobin (Hba1c), lipid profile, kidney function test (serum creatinine and blood urea nitrogen) and the urinary dipstick to search for proteinuria. A cardiologist and an ophthalmologist at the same centre performed an electrocardiogram and ophthalmoscopy. In the newly diagnosed diabetes/prediabetes patients with confirmed DSP, additional infectious (HIV, hepatitis B and C) and metabolic (TSH, Vitamin B12) tests were requested to rule out other common causes of neuropathy. Chronic alcohol consumption was equally searched for in these patients.

### 2.3 Study Instruments

#### 2.3.1 Toronto Clinical Neuropathy Score (TCNS)

TCNS is a clinical tool developed in 2002 for diagnosing distal symmetric polyneuropathy (DSP). Comprising 11 items divided into three sections—symptoms, sensory function tests, and myotactic reflexes—it assesses sensory, motor, and lower‐limb reflex findings commonly evaluated in neuropathy patients [17]. Symptoms such as numbness, tingling, pain, weakness, and ataxia are recorded as present or absent. At the same time, sensory tests, including light touch, pinprick, temperature, position, and vibration, are graded as normal or abnormal at the toes. Knee and ankle reflexes are assessed as normal, reduced, or absent on both sides [7]. The TCNS has a scoring range from 0 to 19, with higher scores indicating greater neuropathy severity, and is interpreted as follows: 0–5 = no neuropathy, 6–8 = mild neuropathy, 9–11 = moderate neuropathy, and ≥ 12 = severe neuropathy [18]. TCNS has been applied in research settings, including studies in patients with type 1 diabetes in Cameroon [19].

#### 2.3.2 Douleur Neuropathique En 4 Points (DN4)

The DN4 questionnaire is a clinical tool designed to assess neuropathic pain by evaluating pain characteristics, associated symptoms, and the presence of hypoesthesia or allodynia. Developed in 2004, the DN4 includes a scoring system where a score of ≥ 4 out of 10 suggests neuropathic pain. This tool was initially validated by analyzing pain characteristics in patients with confirmed central or peripheral nervous system lesions across five centers in France and Belgium [20]. The DN4 has since been used in various settings, including among type 2 diabetes patients in Cameroon, highlighting its clinical utility [21].

#### 2.3.3 Vibration Perception Threshold (VPT)

VPT is a quantitative evaluation of vibration sense, which has shown results superior to those of the classical 128 Hz tuning fork. VPT was evaluated with a biothesiometer device, the VibroSense‐Genesis Medical Systems Pvt. Ltd, Telangana, India. The method of Saha et al. [22] was used in this study. The stimulus was applied with progressively increasing intensity at a frequency of 1 V per second over six points measured in both feet: great toe, 1st metatarsal, 3rd metatarsal, 3rd and 5th metatarsal, instep, and heel. An average was then calculated. The first probe was applied to the patient's hand to explain the early vibration feeling. Then the patient was asked to concentrate on their feet and to tell as soon as they started feeling the vibration, and the value was noted. The grading system used was Normal ≤ 15 V Grade I‐16–25 V Grade II > 25 V. By comparing its performance to the gold standard nerve conduction studies (NCS), the VPT using this method had a sensitivity and specificity of 70% and 86.6%, respectively. Positive and negative predictive values were 84% and 74.3 %, respectively [23].

#### 2.3.4 Electrodiagnostic Studies (EDX)

EDX is considered the gold standard for large‐fibre peripheral nerve disorders in general. The Toronto Consensus on DN of 2010 recommends its use for the diagnosis of definite DSP [6]. As per the recommendations of Yang et al., the identification of distal latencies, CMAPs, SNAPs, CV, and F wave latencies was measured in the upper and lower limbs. Abnormalities on at least two nerves were necessary for the electrophysiological diagnosis of DN [24].

#### 2.3.5 Electrochemical Skin Conductance (ESC) Assessment Using Sudoscan

Sudoscan (Impeto Medical, Paris, France) is a novel instrument that allows for the rapid and non‐invasive evaluation of C fibre innervated sweat glands. A low‐voltage, painless current (< 4 V) is applied to the hands and feet to stimulate sweat glands. A measurement of electrochemical skin conductance expressed in microsiemens for the hands and feet, based on chloride ion concentration, is generated from the derivative current associated with the applied voltage [25]. By comparing ESC results against the gold standard EDX in English DSP patients, Mao et al. reported a sensitivity and specificity of 87.0% and 76.2%, respectively [26]. In non‐diabetic populations, feet ESC results had similar performance for the diagnosis of SFN compared to the intraepidermal nerve fibre density (IENFD), which is considered the gold standard to date [27]. In another study, ESC had a similar diagnostic utility as IENFD based on receiver operating characteristic curve analysis (0.761 and 0.725) for the diagnosis of DSP [28]. The Sudoscan equally has an integrated algorithm that calculates a CAN risk score based on ESC, age, sex, and body mass index (BMI). It had a fairly good diagnostic efficacy compared to cardiac autonomic reflex testing in a Chinese population with an area under the curve of 0.69 [29]. A previous report demonstrated the feasibility and utility of ESC measurement in an African cohort of patients with diabetes [30]. Hands and feet ESC values below 50 and 60 microsiemens were considered abnormal in an African American population [31], and these criteria were used in our study owing to the absence of local normative values. The 2022 international expert consensus recommendations suggest the Sudoscan may be used to assess small fibre function in diabetic patients [3].

### 2.4 Definition of Study Variables

DN was defined and classified according to the Toronto 2010 recommendations. Confirmed DSP was the presence of an abnormality of NCS and symptoms or signs of neuropathy [6]. This corresponds to large fibre neuropathy. SFN was defined as symptoms, clinical signs of small fibre damage, normal sural NCS, and abnormal ESC values. Subclinical DSP was defined as the presence of no signs or symptoms of neuropathy coupled with abnormal NCS or ESC results. Painful DSP was defined as a DN4 score ≥ 4 with a confirmed small or large fibre neuropathy [20]. The Grading system of Dyck et al., recommended by the Toronto Consensus [6], was used for severity assessment: Grade 0, no abnormality of NCS. Grade 1a, abnormality of NCS without symptoms or signs. Grade 1b, NCS abnormality of stage 1a plus neurologic signs typical of DSP but without neuropathy symptoms. Grade 2a, NCS abnormality of stage 1a with or without signs (but if present, < 2b) and with typical neuropathic symptoms. Grade 2b, NCS abnormality of stage 1a, a moderate degree of weakness (i.e., 50%) of ankle dorsiflexion with or without neuropathy symptoms. Diabetic autonomic neuropathy (DAN) was defined by the presence of symptoms of autonomic nervous system involvement, either digestive, cardiovascular, or urogenital, in the absence of a known pathology of those systems [6]. Additionally, CAN was defined by the presence of orthostatic hypotension (a reduction of systolic blood pressure of at least 20 mmHg or diastolic blood pressure of at least 10 mmHg within 3 min of standing) [32] and/or an abnormal CAN risk score given by the Sudoscan [29]. The 50% threshold suggested by Impeto Medical, Paris, France, and integrated into the device was used in the absence of local norms. Treatment‐induced neuropathy of Diabetes (TIDN) was defined by the criteria of Gibbons et al.: The acute onset of pain with or without autonomic symptoms within 8 weeks of intense glucose control with ≥ 2% drop of HbA1c over the past 3 months [33]. Focal neuropathies were defined as either entrapment neuropathies or cranial neuropathies based on the Toronto Expert Panel which classified DN into generalized, focal, and multifocal neuropathies in 2011 [34].

### 2.5 Data Analysis

Data were analyzed using SPSS version 20.0. Pearson's Chi‐squared test was used to compare categorical data. Means of continuous variables were compared between groups with the aid of the one‐way multiple analyses of variance (1‐ANOVA). All statistical tests were two‐sided and considered statistically significant at *p* < 0.05.

Linear regression models were applied to examine the association between the TCNS and diabetes duration for both large and small fiber neuropathy. Confidence intervals were calculated, and plots were generated using R software (version 2023.12.1).

## 3 Results

### 3.1 Demographic and Clinical Characteristics of Participants in the Study Sample (See Table 1)

Eighty‐eight participants were initially enrolled in the study, but four were excluded due to incomplete data during the analysis phase (no final diagnosis). The final study population consisted of 84 participants, of whom 77 (91.7%) had type 2 diabetes mellitus, 2 (2.4%) had type 1 diabetes mellitus, and 5 (6%) had glucose intolerance. There were 32 females (38.1%) with a male/female sex ratio of 1.63:1. The mean age at diagnosis and disease duration were 56.98 ± 10.77 years and 7.73 ± 8.55 respectively. The mean Hba1c during the study was 7.82 ± 1.92.

**TABLE 1:** Demographic and clinical characteristics of the study sample.

	Total patients	Large fibre neuropathy	Small fibre neuropathy	*p* value
*N*	84	32	21	—
Sex = *M* (%)	52 (61.9)	22 (68.8)	9 (42.9)	0.09
Age (years) (mean (SD))	56.98 (10.77)	61 (10.11)	55.29 (9.91)	**0.048**.
Body mass index (BMI) (mean (SD))	28.88 (4.96)	29.19 (5.36)	27.67 (5.65)	0.33
Pre‐diabetes/glucose intolerance (*n* (%))	5 (6)	1 (3.1)	3 (14.3)	—
Diabetes type (*n* (%))				
Type 1	2 (2.4)	0 (0.0)	1 (4.8)	—
Type 2	77 (91.7)	31 (96.9)	17 (81)	0.13
Diabetes duration (mean (SD))	7.73 (8.55)	7.59 (6.83)	7.80 (10.94)	0.94
HbA1C (mean (SD))	7.8 (1.85)	8.37 (1.82)	7.35 (2.22)	0.08
Toronto clinical neuropathy score (TCNS) (mean (SD))	5.54 (4.51)	8.66 (4.60)	3.9 (3.22)	**< 0.001**
Neuropathy pain score, douleur neuropathique 4 questions (DN4) (mean (SD))	1.81 (1.4)	2.50 (1.50)	1.55 (1.27)	**0.047**
Abdominal circumference (mm) (mean (SD))	106.1 (12.45)	109.94 (12.37)	97.67 (9.25)	**0.016**
Systolic blood pressure (mmHg) (mean (SD))	130.7 (15.22)	136.1 (17.92)	121.17 (13.74)	**0.03**
Diastolic blood pressure (mmHg) (mean (SD))	79.48 (10.83)	79.92 (14.26)	79.75 (9.96)	0.97
Mean total cholesterol (g/L) (mean (SD))	1.95 ± 0.52	2.05 (0.57)	1.65 (0.43)	0.053
Mean LDLc (g/L) (mean (SD))	1.25 ± 0.50	1.38 (0.43)	1.01 (0.39)	**0.005**
Mean HDLc (g/L) (mean (SD))	0.47 ± 0.19	0.43 (0.19)	0.53 (0.21)	0.11
Mean Triglycerides (g/L) (mean (SD))	1.06 ± 0.75	1.07 (0.52)	0.89 (0.54)	0.40
Vibration perception threshold (VPT) Lower limbs (mean (SD))	9.83 (7.55)	14.94 (9.57)	7.86 (3.99)	**< 0.001**
Electrochemical skin conductance (ESC) Upper limbs (mean (SD))	62.08 (12.64)	63.31 (12.8)	54.95 (14.53)	**0.032**
Electrochemical skin conductance (ESC) Lower limbs (mean (SD))	61.65 (14.23)	54.75 (16.9)	58.71 (10.71)	0.3
Autonomic cardiac neuropathy risk score (mean (SD))	36.12 (11.7)	40.0 (12.14)	33.19 (11.63)	**0.048**
Sural SNAP, μV (mean (SD))	8.46 (3.44)	4.7 (1.55)	11.27 (2.37)	**0.043**
Sural nerve CV, m/s (mean (SD))	49.88 (8.47)	48.7 (0.28)	48.6 (4.16)	0.97
Classification Dyck (%)				
1a	—	3 (9.4)	0 (NaN)	—
1b	—	4 (12.5)	0 (NaN)	—
2a	—	23 (71.9)	0 (NaN)	—
2b	—	2 (6.3)	0 (NaN)	—
Carpal tunnel syndrome CTS (%)	27 (32.14)	10 (31.25)	7 (33.3)	0.88
Symptomatic (%)	16 (19.04)	6 (18.75)	4 (19.05)	0.97
Bilateral (%)	20 (23.8)	7 (21.88)	6 (28.57)	0.59

*Note:* Values presented in bold indicate statistical significance (*p* < 0.05).

A large proportion of the study sample, 76 participants (90.5%), had one or more vascular risk factors, mostly dyslipidemia (70.2%), obesity (53.6%) and hypertension (47.6%). The most frequent chronic complication was ischemic heart disease, present in 12 participants (14.3%).

### 3.2 Prevalence and Types of DN (See Figure 1)

The prevalence of DN in all forms included was 86.9% (73/84). The different subtypes identified in the study sample were DSP (63.1%); DAN (40.5%); mononeuropathies (36.9%); asymmetric sensory axonal neuropathies (4.8%); and TIND (1.2%). Only five participants (7.0%) with DSP had a DN4 score ≥ 4 and thus met the criteria for painful DSP. Four participants in the DSP (4.8%) group had a subclinical neuropathy. Approximately one‐third of the participants with DSP, representing 25.0% of the whole study sample, had a pure small fibre neuropathy.

**FIGURE 1:**
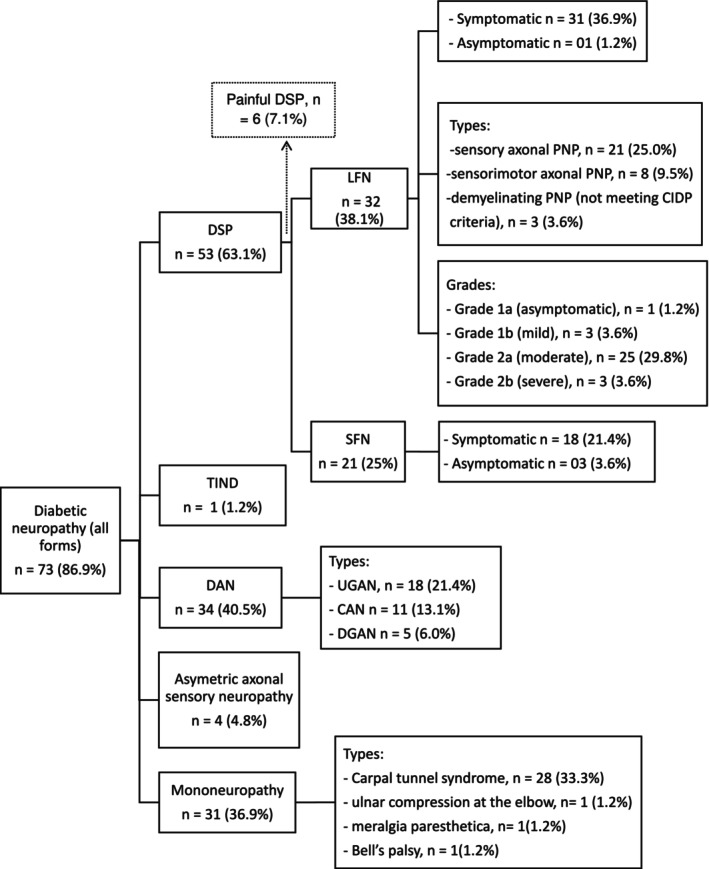
Graphical summary of the distribution of diabetic neuropathy subtypes in the study population. DSP, Distal symmetric sensorimotor polyneuropathy; DAN, Diabetic autonomic neuropathy; UGAN, Urogenital autonomic neuropathy; CAN, Cardiac autonomic neuropathy; DGAN, Digestive autonomic neuropathy; TIND, Treatment‐induced neuropathy of Diabetes; SFN, small fibre neuropathy; PNP, polyneuropathy; CIDP, Chronic inflammatory demyelinating polyneuropathy.

The most frequent finding in nerve conduction studies was sensory axonal polyneuropathy (21 participants on 84, 25.0%). Three subjects (3.6%) had primarily demyelinating polyneuropathy, which did not meet the criteria for chronic inflammatory demyelinating polyneuropathy (CIDP). Twenty‐five (25) patients with NCS (29.8%) were classified as grade 2a according to the criteria of Dyck et al. (see Figure 1 for NCS details). DAN was found in 34 subjects (40.5%), mostly with urogenital symptoms (25.3%). We identified 31 participants (36.9%) with mononeuropathy, most of which were carpal tunnel syndrome (28 participants, 33.3%, with 12 being asymptomatic). Bell's palsy and meralgia paresthetica both had a prevalence of 1.2% (1 participant). In both cases, the neuropathy led to the diagnosis of DM.

The mean TCNS score in the whole study population was 5.54 ± 4.51, while 39 participants (46.4%) had scores defining DSP. Thirty‐three (33) out of fifty‐three (53) participants with confirmed DSP had a TCNS score > 5. Only 8 of the 21 participants (38.1%) with electrophysiological SFN had pathological TCNS values.

### 3.3 Factors Associated With Diabetic Neuropathy in the Study Cohort (See Figure 2)

The scatter plot with linear regression showed a trend of milder disease severity and shorter diabetes duration in SFN compared to LFN (though not statistically significant). This trend was supported by a higher proportion of SFN in the prediabetes group (60.0%) compared to the diabetes group (22.7%), though not statistically significant (*p* = 0.06). The prevalence of LFN was higher in the diabetes group (39.2%) compared to those with glucose intolerance 22.7%. The difference was not statistically significant (*p* = 0.39).

**FIGURE 2:**
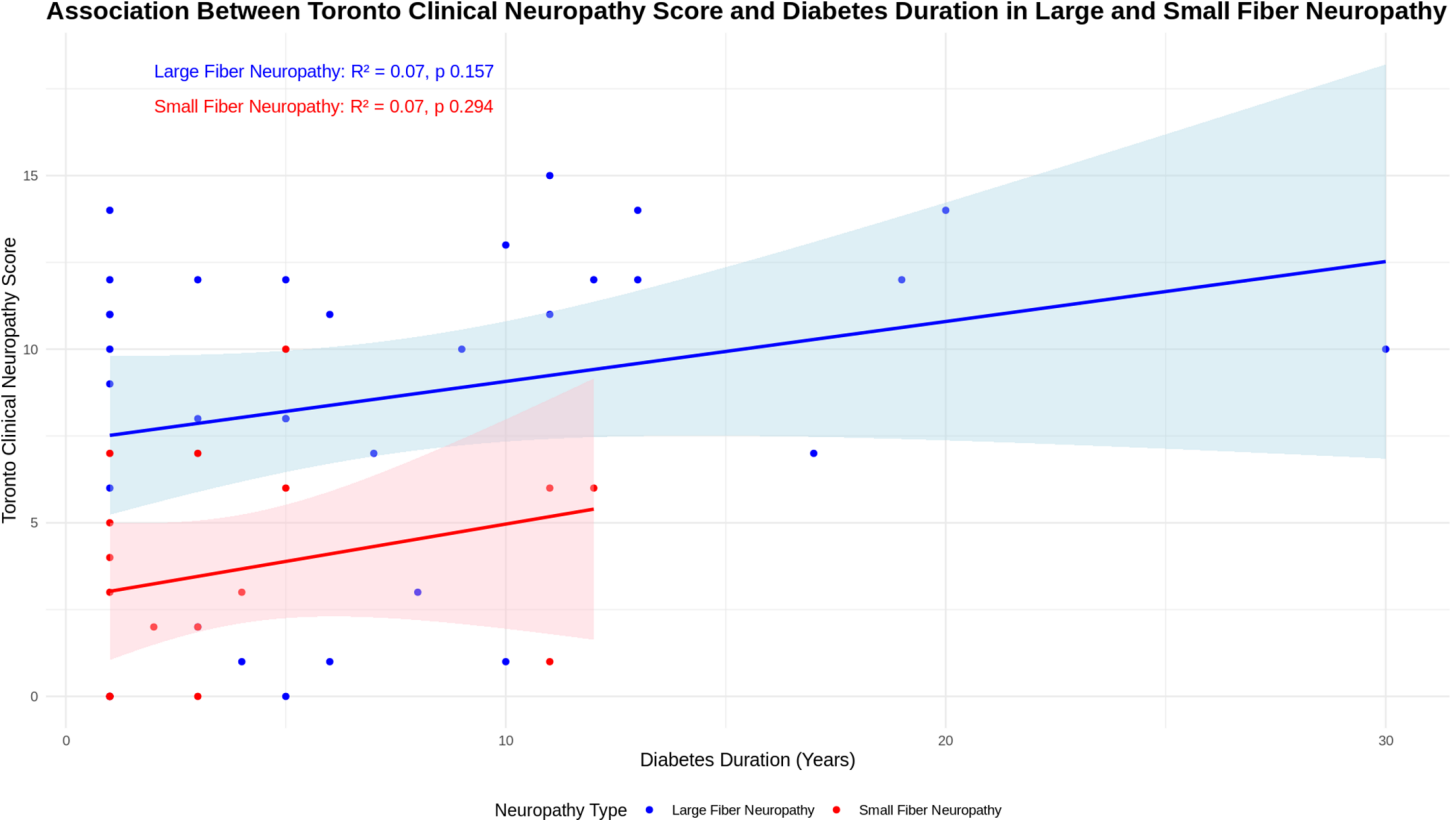
Association between TCNS and diabetes duration in large and small fibre neuropathy.

Age, systolic blood pressure, LDL cholesterol levels, and abdominal circumference were higher in participants with LFN compared to SFN on univariate analysis (see Table 1).

## 4 Discussion

This study aimed to identify the prevalence of confirmed DN and its various clinical and electrophysiological subtypes in an African cohort. There is no report of certain DN subtypes in sSA, to the best of our knowledge. We found that the prevalence of all forms of DN was 86.9%. Reported prevalence in the literature varies widely, from 0.58% to 88.7% [35, 36]. This heterogeneity arises from variations in diagnostic criteria, study populations, and ethnic backgrounds. In most studies globally, particularly in sSA, a purely clinical approach is employed for diagnosing DN [4, 37, 38]. Consequently, many of these studies focus solely on diagnosing DSP, overlooking other forms of DN. Furthermore, subclinical neuropathies are often not identified in such research.

The prevalence of DSP subtype in our study was 63.1%, with subclinical forms going as high as 5.0%. To our knowledge, this is the first study reporting the prevalence of definite DSP in sSA, according to the Toronto Consensus for DSP. Interestingly, 46.4% of our study population had a clinical diagnosis of DSP (TCNS > 5), a prevalence very similar to that reported by a recent meta‐analysis of several African studies (46.0%) [4]. An earlier study in a different setting in Cameroon in 2015 reported a prevalence of 33.3% of DN using a Diabetic Neuropathy Examination score of > 3/16 and/or a monofilament score of < 5/9 [39]. The prevalence of DN in a recent study in Ukraine that equally included neurophysiological data for the diagnosis was as high as 79.55% [36]. Our data suggest that the pure clinical approach might underestimate the true prevalence of DSP in African cohorts. A previous study in which neuromuscular experts diagnosed DSP based only on symptoms and signs had shown that the disease was underdiagnosed in close to 40% of cases in comparison with EDX diagnosis [7]. Several others have emphasized this underdiagnosis even with standardized clinical scores, including the Michigan Neuropathy Screening Instrument (MNSI), the Toronto Clinical Neuropathy Score (TCNS) and the United Kingdom Screening Test [18, 40, 41].

A very significant feature in this study is the prevalence of pure small‐fibre neuropathy, which was 25.0%, representing over 1/3 of all patients with DSP. To our knowledge, this has never been reported in an African cohort. There are few published studies evaluating the comparative proportion of SFN and LFN in cohorts of patients with diabetes. In a Danish study including type 2 diabetic patients, the authors reported a variable prevalence of small and large fibre neuropathy between 1.4%–13.1% for SFN and 9.3%–21.5% for LFN, depending on the criteria (clinical and/or electrophysiological) applied. Across the four models tested, SFN represented a maximum of 11.1% of the DSP subtypes [42]. In a multicentric Italian study including 816 diabetic patients, Truini et al. reported a prevalence of 36% for DSP and 2.5% for SFN [43]. There is increasing data on racial variability in small fibre function in diabetic patients. A comparative study of corneal nerve fibre length, corneal nerve branch density, and heart rate variability between South Asians and European patients with diabetes in the UK reported, for example, a significantly better‐preserved small fibre function in the first group [44]. Our findings might therefore suggest a higher frequency of pure small fibre dysfunction in populations of African descent. The reasons might be acquired (variability in BMI, triglyceride levels, smoking) or due to ethnic variability in sodium and potassium channel genes, as has been shown mainly in cardiac diseases. This would need to be confirmed in larger studies with more heterogeneous populations. This high prevalence is nevertheless worth noting, as several studies suggest the possibility of complete reversibility at this stage. In a large German cohort including 920 patients recently diagnosed (< 1 year) with type 1 and type 2 diabetes, the authors found significantly more small fibre pathology (21.8% of abnormal IENFD) than large fibre dysfunction (11.8% of abnormal sural SNAP) at baseline. Interestingly, after 4 years of follow‐up, there was a reversal to some degree in both groups, but the proportion of return to normal values was much higher for IENFD (8.7%) than for sural SNAP (3.7%) [45]. Hence the proportion of patients with pure SFN in our cohort might represent the subgroup with the highest potential benefit from lifestyle modifications for metabolic and cardiovascular risk factor control, as suggested by Umapathi et al. [46]. We had earlier reported the case of a patient from our cohort diagnosed with SFN due to glucose intolerance. After 7 months of follow‐up with lifestyle modifications and vascular risk factor control, the patient was symptom‐free and had normal ESC values [47]. This underscores the need for SFN identification in our context despite technological limitations. Several authors have recently advocated for a paradigm shift towards early diagnosis of DSP at the stage of pure SFN, similar to retinal assessments for retinopathy and microalbuminuria for nephropathy, as these measures have transformed the outcomes of diabetic nephropathy and retinopathy, respectively [48]. If the results of this study are reproduced in other settings, this approach might be even more pertinent in African populations, with health policy implications. This paradigm shift would require the inclusion of more appropriate tools for both screening and diagnosis of DSP for better management. Our cohort's moderate sensitivity of clinical testing was largely attributed to the SFN group. More than 50% (13/21) of the participants with SFN had TCNS scores < 5. Devigili et al. reported the poor performance of clinical evaluation alone in SFN [49].

It is equally worth noting that participants with large‐fibre diabetic sensorimotor polyneuropathy (DSP) were significantly older and had higher systolic blood pressure, LDL cholesterol levels, and abdominal circumference, all established risk factors for DN. This observation aligns with previous studies suggesting a progression in disease severity from small‐ to large‐fibre dysfunction [48]. Moreover, the increasing prevalence of non‐communicable diseases (NCDs), such as diabetes in sub‐Saharan Africa, driven by lifestyle changes and environmental factors, underscores the urgency of addressing these risk factors to mitigate the burden of DN in the region [50].

The subgroup of patients with sub‐clinical neuropathy (4.8%) is equally of particular interest, as studies have shown that 25% of patients with subclinical DSP developed clinical manifestations over a 4‐year follow‐up period [51]. An equal proportion of participants (4.8%) presented a non‐length‐dependent axonal sensory neuropathy. This is important to note as this profile suggests a sensory neuronopathy, which should prompt investigation into other causes, mainly paraneoplastic and autoimmune [52].

To the best of our knowledge, TIND has never been reported in an African cohort. Though rare (1 patient in our cohort, 1.2%), our findings should prompt the attention of endocrinologists and neurologists practicing in sSA to consider its existence. Tran et al. emphasized risk awareness (by health personnel) as the key to the prevention of TIND [53].

The prevalence of DAN (40.5%) was lower than reported in a previous study from Burkina Faso (58.6%) [31]. Interestingly, cardiac autonomic neuropathy (CAN) was the rarest form of DAN identified in that cohort (2.6%). This may be because CAN was identified only by measuring orthostatic hypotension, which occurs mainly at advanced stages of the disease [54]. In the present study, CAN (13.1%) was diagnosed using a cardiac risk score calculated by an integrated algorithm in the SUDOSCAN Impeto medical device. The Sudoscan might therefore represent an alternative to a purely clinical approach for diagnosing CAN in resource‐limited settings. This is important considering the high 5‐year mortality (up to 50%) of advanced CAN [55].

Focal neuropathies were included as complications of diabetic neuropathies based on the classification of the Toronto Consensus Panel into generalized, focal, and multifocal neuropathies [34]. Several authors have reported an increased prevalence of cranial and entrapment neuropathies in diabetic patients, mainly carpal tunnel syndrome, often bilateral and asymptomatic, as in our sample [2, 56]. Upton et al. proposed the “double crush” hypothesis: endoneurial ischemia and metabolic derangements due to diabetes made peripheral nerves more susceptible to a second hit at entrapment sites [57]. However, we had no specific means of confirming the role of diabetes in participants who presented with entrapment neuropathies. Excluding these, the prevalence of DN in our sample drops to 73.8%, as 11 out of the 31 participants with focal neuropathies did not have an associated generalized neuropathy.

This study acknowledges limitations, primarily the absence of a gold standard IENFD or QST for SFN diagnosis, potentially inflating the observed prevalence. Given the scarcity of these modalities in our setting and across sub‐Saharan Africa (sSA), electrochemical skin conductance (ESC) was used as a surrogate. While ESC correlates with IENFD [22, 58] and is supported in diabetic neuropathy guidelines [3], a recent review advises against its sole use in place of skin biopsies [59]. Therefore, our findings require validation in a larger cohort employing IENFD or QST. Notably, all SFN patients in our sample presented with normal nerve conduction studies (NCS), consistent with NEURODIAB criteria for probable SFN [60]. Further limitations include a small, single‐center sample, potentially biased towards affluent, insured patients, thus limiting generalizability. However, demographic and clinical characteristics were comparable to a larger tertiary center study [61], despite a lower mean Hba1c (7.8% vs. 8.6%). Finally, both small fiber and autonomic neuropathy assessments relied solely on symptom reporting, precluding the use of standardized tests like COMPASS 31, which would have enhanced diagnostic accuracy.

## 5 Conclusion

This study, which combined clinical and electrophysiological tools, reports a very high prevalence of DN in an sSA Cohort. The electrophysiological approach allowed us to describe, for the first time to the best of our knowledge, various subtypes of neuropathies which are usually englobed under the umbrella of DSP: small fibre neuropathy, treatment‐induced neuropathy of diabetes, and sensory neuronopathies. The prevalence of SFN might be higher in African subjects than in other races, and this may have management and policy implications. Further studies on more extensive and more heterogeneous samples, ideally using instruments with more robust validation data such as IENFD and QST, are necessary to better describe the specificities of DN in sSA. Our results suggest the adoption of a different approach for diagnosing and characterizing DN both in clinical and research settings.

## Author Contributions

S.E.C., G.L., U.N.T., A.K.N. (Conception). S.E.C., G.L. A.T.A., U.N.T., A.K.N. (Design). S.E.C., G.L., L.N., H.N.T.K., R.J.N., G.S., L.P. (Data collection). S.E.C., J.F.I.R., A.T.A., U.N.T., A.K.N. (Data analysis and interpretation); S.E.C., G.L., J.F.I.R., and A.T.A. wrote the first draft, and all authors critically reviewed and approved the final version of the manuscript. U.N.T. and A.K.N. took the final decision to submit.

## Ethics Statement

Ethical clearance for the study was obtained from the Research and Ethics Committee of Jordan Medical Services (Registration Number, 2021/12/29).

## Consent

All participants gave informed consent to participate in the study.

## Conflicts of Interest

The authors declare no conflicts of interest.

## Data Availability

The data that support the findings of this study are available on request from the corresponding author. The data are not publicly available due to privacy or ethical restrictions.
